# A new way to read the price: measuring global affordability of selected NOACs

**DOI:** 10.3389/fphar.2026.1767958

**Published:** 2026-04-29

**Authors:** Julia Cynar, Afonso Miguel Cavaco, Elżbieta Nowakowska, Krzysztof Kus, Piotr Ratajczak, Tomasz Zaprutko

**Affiliations:** 1 Department of Pharmacoeconomics and Social Pharmacy, Poznan University of Medical Sciences, Doctoral School, Poznan, Poland; 2 Faculdade de Farmácia, Universidade de Lisboa, Lisbon, Portugal; 3 Collegium Medicum University of Zielona Gora, Zielona Gora, Poland; 4 Department of Pharmacoeconomics and Social Pharmacy, Poznan University of Medical Sciences, Poznan, Poland

**Keywords:** affordability, Big Mac index, cross country comparison, drug, drug index, drug pricing, international dollar (Int$), NOAC

## Abstract

**Objectives:**

Access to medicines is an important component of health systems, yet their affordability differs across countries. Novel oral anticoagulants are widely used in the prevention and treatment of thromboembolic disorders, but their prices may vary between national markets. This study aimed to develop and test a methodological approach for comparing the prices of selected NOACs across countries using purchasing power parity (PPP) adjustments.

**Methods:**

Retail prices for Pradaxa®, Xarelto®, and Eliquis® were collected. The analysis covered 12 countries. Prices were expressed in nominal US$ and PPP-adjusted International Dollars (Int$). Drug-specific indexes inspired by the Big Mac Index were calculated to compare the exchange rate implied by drug prices with the PPP exchange rate, using the United States as the reference market.

**Results:**

The United States showed the highest prices for all three medicines in both nominal and PPP-adjusted comparisons. Lower prices were observed across the analysed European countries. Drug index calculations showed all countries’ prices were undervalued relative to the United States, ranging from −58.52% to −87.19%

**Conclusion:**

Combining PPP-adjusted prices expressed in International Dollars with drug-specific indexes offers an additional perspective for international medicine price comparisons and helps interpret differences in relative price levels across markets.

## Introduction

According to the World Health Organization (WHO) Constitution access to essential medicines is a fundamental human right (Constitution of the World Health Organization, 1946). Inextricably linked to this is the cost of the medication, which is a crucial factor shaping access to treatment (Constitution of the World Health Organization, 1946). Consequently, ensuring the accessibility of affordable medications to the population becomes a crucial objective of drug policies.

Considering a range of diseases highlighted as significant for humans in the first half of the 21st century, caused, e.g., by longer life expectancy and lifestyle, cardiovascular disorders have become very important among many other ailments. This results in a significant increase in the importance of medications from the NOACs (Novel Oral Anticoagulant) group (Godman et al., 2018). It is in line, for instance, with the data presented by WHO where the average annual number of deaths caused by cardiovascular events reaches 17.9 million each year (Cardiovascular Diseases, n.d.). NOACs are anticoagulants used in the prevention and treatment of blood clots. They are commonly used in conditions such as atrial fibrillation, deep vein thrombosis (DVT) and pulmonary embolism (PE) (Eliquis | European Medicines Agency (EMA) 2018; Pradaxa | European Medicines Agency, 2024; Xarelto | European Medicines Agency, 2021).

This study takes a novel approach to comparing medicine prices across countries, using selected NOACs as an exemplar to explore broader patterns of international price variation and cross-country price comparability. It focuses on three drugs - Pradaxa® (dabigatran) (Pradaxa | European Medicines Agency, 2024), Xarelto® (rivaroxaban) (Xarelto | European Medicines Agency, 2021) and Eliquis® (apixaban) (Eliquis | European Medicines Agency EMA, 2018) - selected for their increasing market relevance and status as original brand-name drugs. Using purchasing power parity adjusted price expressed in International Dollars (Int$) for comparison and adapting the Big Mac Index to create drug-specific indexes (e.g., “Pradaxa Index”). The study aims to assess and compare the real-world prices of selected NOACs across different countries and to highlight international price disparities that may affect patients’ access to treatment, with the primary objective of developing and testing a methodological approach applied to a sample of 12 countries using purchasing power parity (PPP) adjustments. The analysis focuses on full retail prices (100% out-of-pocket payments) and uses Int$ and Drug Index based on Big Mac Index to enable cross-country comparability.

## Methods

The costs of the drugs considered in the study were based on retail prices reflecting 100% out-of-pocket payments. For consistency and transparency, the selection of these medications was based on the same dosage, number of tablets per package, and pharmaceutical company. This approach minimized variability related to local substitution practices, generic availability allowing for a direct and methodologically uniform cross-country comparison of retail prices. The analysis included 12 countries for Xarelto® (dosage: 20 mg, package size: 28 tablets), Eliquis® (dosage: 2.5 mg, package size: 60 tablets) and Pradaxa® (dosage: 110 mg, package size: 60 tablets). These presentations were chosen as commonly marketed and widely available strengths across countries, ensuring feasibility and comparison and consistent data collection. The analysis focused on Pradaxa®, Xarelto® and Eliquis® because they were first commercially available NOAC drugs and have played a key role in improving the standard of treatment for thromboembolic diseases.

Data were obtained from official websites for the Czech Republic (Veřejnost, n.d.), Iceland (Lyfjaverðskrá, n.d.), Norway (DMPs Legemiddelsøk oppgraderes, n.d.), Denmark (Medicinpriser.Dk, n.d.), Portugal (Pesquisa medicamento, n.d.), Sweden (Tandvårds-Läkemedelförmånsverket - Tandvårds- och läkemedelsförmånsverket TLV, n.d.), France (Page d’accueil, n.d.), Ireland (HSE - PCRS Search Reimbursable Items, n.d.), Netherlands (Nederland, Zorginstituut, n.d.), Latvia (Sākumlapa En, Nacionālais Veselības Dienests, n.d.). In Poland, the analysed medicines (Xarelto 20 mg 28 tab, Pradaxa 110 mg 60 tab, Eliquis 2.5 mg 60 tab) were not included in the national coverage schemes during the study period. Therefore, retail pharmacy prices were obtained from a professional drug information database (Leki - indeks preparatów - Indeks24.pl - wyszukiwarka i baza leków, n.d.) used by healthcare professionals, which reflects actual market prices and full out-of-pocket costs. For United States the data were directly obtained from pharmacies. Retail prices were sourced from three community pharmacies and averaged, data was obtained via our relatives and friends who live in this country. The data on medication prices was collected from January 15 to 30 November 2023. Participation in price reporting for the United States was voluntary, anonymised and solely for scientific purposes.

To compare medication prices, both nominal prices in US$ and International Dollar (Int$), with Int$ reflecting purchasing power parity (PPP) to account for differences in living costs and income. Currency conversions were based on 2023 World Bank exchange rate (World Development Indicators | The World Bank, n.d.), ensuring comparability with the period in which the medication prices were collected. Additionally, drug specific indexes for Pradaxa®, Xarelto®, and Eliquis® based on the Big Mac Index were developed.

The Big Mac Index serves as a measure of purchasing power parity (PPP) between currencies (The Economist, n.d.). The Big Mac Index is based on the theory of purchasing power parity, suggesting that exchange rates should adjust over time to equalize prices of identical goods and services in different countries. The index is calculated by taking the price of a Big Mac in each country and converting it to US dollars using the current exchange rate. This provides an approximate assessment of the relative value of the local currency. By comparing Big Mac prices in different countries, the index can help identify overvalued or undervalued currencies (The Economist, n.d.).

Similarly, the Pradaxa, Xarelto, and Eliquis Indexes, like the Big Mac Index, are based on the theory of purchasing power parity. These indexes are conceptually inspired by the Big Mac Index but adapted to pharmaceuticals. Unlike Int$, which represents the adjusted price level, the Drug Index evaluates whether a drug’s price in a given country aligns with the purchasing power implied by PPP relative to the reference market (the United States). A Drug Index value of zero indicates that the local price follows the expected PPP relationship. Negative values indicate that the drug is priced below the PPP-based benchmark, while positive values would indicate overvaluation. The Drug Indexes were computed based on the price of each medication in each country, which is then converted to Int$ using the exchange rate provided by the World Bank (World Development Indicators | The World Bank, n.d.). In summary, the Int$ and drug indexes provide complementary tools for comparing drug prices across countries while accounting for differences in purchasing power.

Drug index formula:

$$Drug\;Index=\frac{{\;}^{\#}Implied\;PPP\;exchange\;rate-Actual\;PPP\;Exchange\;Rate}{Actual\;PPP\;Exchange\;Rate}*100\%$$

$${\;}^{\#}Implied\;PPP\;exchange\;rate=\frac{price\;of\;the\;drug\;\left(local\;currency\right)}{price\;od\;the\;drug\;in\;the\;USA\;\left(\mathrm{Int}\$\right)}$$

## Results

For Pradaxa the lowest PPP–adjusted (Int$) prices were observed in the Netherlands, while for Xarelto and Eliquis the lowest price (Int$) was recorded in Sweden. The highest drug prices were observed in the United States (United States), with values of Int$ 531.79 for Pradaxa®, Int$ 542.92 for Xarelto®, and Int$ 599.97 for Eliquis®. Across the three NOACs analysed, the United States consistently exhibited the highest nominal and PPP-adjusted prices. The lowest nominal prices were observed in Poland for all analysed drugs.

Drug-specific indexes indicated that all currencies were undervalued relative to the United States, which served as the reference country. Detailed prices and index results are presented in Tables 1–6.

**TABLE 1:** Prices of Pradaxa^®^ in local currencies, Int$ and US$.

Country	Price (local currency, LC)	Actual PPP exchange rate (LC/Int$)	Price (Int$)	Official exchange rate (LC/US$)	Price (US$)
Netherlands	56.40	0.74	76.22	0.92	61.30
Sweden	655.19	8.51	76.99	10.61	61.75
Norway	740.50	8.89	83.30	10.56	70.12
France	57.23	0.67	85.42	0.92	62.21
Ireland	76.01	0.75	101.35	0.92	82.62
Iceland	14318.00	140.16	102.15	137.94	103.80
Poland	198.00	1.88	105.32	4.20	47.14
Denmark	757.81	6.11	124.03	6.89	109.99
Portugal	69.86	0.52	134.35	0.92	75.93
Czech Republic	1784.35	12.55	142.18	22.20	80.38
Latvia	87.15	0.50	174.30	0.92	94.73
United States	531.79	1.00	531.79	1.00	531.79

**TABLE 2:** Prices of Xarelto^®^ in local currencies, Int$ and US$.

Country	Price (local currency, LC)	Actual PPP exchange rate (LC/Int$)	Price (Int$)	Official exchange rate (LC/US$)	Price (US$)
Sweden	616.05	8.51	72.39	10.61	58.06
Poland	145.00	1.88	77.13	4.20	34.52
France	53.44	0.67	79.76	0.92	58.09
Norway	722.90	8.89	81.32	10.56	68.46
Netherlands	60.20	0.74	81.35	0.92	65.43
Iceland	13463.00	140.16	96.05	137.94	97.60
Ireland	74.85	0.75	99.80	0.92	81.36
Denmark	624.68	6.11	102.24	6.89	90.67
Latvia	59.29	0.50	118.58	0.92	64.45
Portugal	65.58	0.52	126.12	0.92	71.28
Czech Republic	1712.46	12.55	136.45	22.20	77.14
United States	542.92	1.00	542.92	1.00	542.92

**TABLE 3:** Prices of Eliquis^®^ in local currencies, Int$ and US$.

Country	Price (local currency, LC)	Actual PPP exchange rate (LC/Int$)	Price (Int$)	Official exchange rate (LC/US$)	Price (US$)
Sweden	653.97	8.51	76.85	10.61	61.64
Norway	772.10	8.89	86.85	10.56	73.12
France	58.95	0.67	87.99	0.92	64.08
Netherlands	67.20	0.74	90.81	0.92	73.04
Poland	172.00	1.88	91.49	4.20	40.95
Iceland	13850.00	140.16	98.82	137.94	100.41
Ireland	76.00	0.75	101.33	0.92	82.61
Denmark	644.13	6.11	105.42	6.89	93.49
Portugal	71.19	0.52	136.90	0.92	77.38
Czech Republic	2044.61	12.55	162.92	22.20	92.10
Latvia	124.42	0.50	248.84	0.92	135.24
United States	599.97	1.00	599.97	1.00	599.97

**TABLE 4:** Pradaxa index: valuation of Pradaxa^®^.

Pradaxa index				
Country	Implied PPP exchange rate	Actual PPP exchange rate	Drug index (%)	Status
Netherlands	0.11	0.74	−85.67%	UV*
Sweden	1.23	8.51	−85.52%	UV
Norway	1.39	8.89	−84.34%	UV
France	0.11	0.67	−83.94%	UV
Ireland	0.14	0.75	−80.94%	UV
Iceland	26.92	140.16	−80.79%	UV
Poland	0.37	1.88	−80.20%	UV
Denmark	1.43	6.11	−76.68%	UV
Portugal	0.13	0.52	−74.74%	UV
Czech Republic	3.36	12.55	−73.26%	UV
Latvia	0.16	0.50	−67.22%	UV
United States	1.00	1.00	0.00%	-

*UV, undervalued.

**The United States, is the reference market; therefore, its Drug Index is set to 0%, and no valuation is assigned.

Calculations for Pradaxa Index in Portugal - example.

Price of Pradaxa in local currency: 69.86 (Table 1).

Price of Pradaxa in the United States (Int$) – reference market: 531.79 (Table 1).

Actual exchange rate (provided by World Bank data): 0.52 (Table 4).

# $\text{Implied PPP Exchange Rate}=\frac{\text{Price}\;\text{in}\;\text{local}\;\text{currency}}{\text{Price}\;\text{in}\;\text{USA}\;\left(\mathrm{Int}\$\right)}=\frac{69.86}{531.79}=0.13$

Drug Index $=\frac{\text{\#Implie}d\;\text{PP}P\;\text{exchang}e\;\text{rat}e-\text{Actua}l\;\text{PP}P\;\text{Exchang}e\;\text{Rat}e}{\text{Actua}l\;\text{PP}P\;\text{Exchang}e\;\text{Rate}}=\frac{0.13-0.52}{0.52}$ * 100% = - 74,74%.

**TABLE 5:** Xarelto index: valuation of Xarelto^®^.

Xarelto index				
Country	Implied PPP exchange rate	Actual PPP exchange rate	Drug index (%)	Status
Sweden	1.13	8.51	−86.67%*	UV
Poland	0.27	1.88	−85.79%	UV
France	0.10	0.67	−85.31%	UV
Norway	1.33	8.89	−85.02%	UV
Netherlands	0.11	0.74	−85.02%	UV
Iceland	24.80	140.16	−82.31%	UV
Ireland	0.14	0.75	−81.62%	UV
Denmark	1.15	6.11	−81.17%	UV
Latvia	0.11	0.50	−78.16%	UV
Portugal	0.12	0.52	−76.77%	UV
Czech Republic	3.15	12.55	−74.87%	UV
United States	1.00	1.00	0.00%	-

*UV, undervalued.

**The United States, is the reference market; therefore, its Drug Index is set to 0%, and no valuation is assigned.

**TABLE 6:** Eliquis index: valuation of Eliquis^®^.

Eliquis index				
Country	Implied PPP exchange rate	Actual PPP exchange rate	Drug index (%)	Status
Sweden	1.09	8.51	−87.19%*	UV
Norway	1.29	8.89	−85.52%	UV
France	0.10	0.67	−85.34%	UV
Netherlands	0.11	0.74	−84.86%	UV
Poland	0.29	1.88	−84.75%	UV
Iceland	23.08	140.16	−83.53%	UV
Ireland	0.13	0.75	−83.11%	UV
Denmark	1.07	6.11	−82.43%	UV
Portugal	0.12	0.52	−77.18%	UV
Czech Republic	3.41	12.55	−72.85%	UV
Latvia	0.21	0.50	−58.52%	UV
United States	1.00	1.00	0.00%	-

*UV, undervalued.

**The United States, is the reference market; therefore, its Drug Index is set to 0%, and no valuation is assigned.

## Discussion

This analysis revealed price variation in selected NOACs across 12 countries. In particular, the United States consistently exhibited the highest nominal and PPP-adjusted (Int$) prices across all three products. Although Int$ prices already reflect differences in purchasing power between countries, our Drug Index provides an additional layer of interpretation. Unlike Int$, which indicates the real cost of a medicine after accounting for local purchasing power, the Drug Index compares the price-implied exchange rate with the actual PPP exchange rate. In our study, the Drug Index highlighted a consistent structural pattern across selected NOACs: Pradaxa ranged from –67.22% to −85.67%, Xarelto from −74.87% to −86.67%, and Eliquis from −58.52% to −87.19%, confirming that the United States represents a structurally higher price level. These findings are consistent with previous studies reporting higher drug prices in the United States compared with European countries (Differences in Costs of and Access to Pharmaceutical Products in the EU | Think Tank | Parlament Europejski, 2011; Kanavos et al., 2013; Kesselheim et al., 2016).

Several structural factors may explain the observed price differentials between United States and European countries. In many European countries, medicine prices are centrally negotiated with public payers, which may increase their bargaining power relative to manufacturers. Pricing frameworks often include formal evaluations, such as health technology assessment (HTA), to assess clinical benefit and cost-effectiveness, and external reference pricing (ERP) to benchmark prices across countries (Rémuzat et al., 2015). In contrast, the United States relies on a decentralized, market-based approach. Manufacturers largely set list prices independently, while subsequent negotiations occur separately with multiple payers, including private insurers, pharmacy benefit managers, and public programs such as Medicare Part D. This fragmented system limits national-level bargaining power. This fragmented structure may limit bargaining power and may partially explain the systematically highest prices observed in our analysis (Syversen et al., 2024; Rodwin, 2021).

However, substantial variation was also observed within Europe. For instance, Poland shows the lowest nominal prices, however, after adjusting the prices for purchasing power parity, by converting prices to Int$, Poland was no longer the least expensive country. This difference is particularly evident for Pradaxa. Although, the nominal price of this medicine is the lowest in Poland, after PPP adjustment it ranks seventh among the 12 analysed countries. Conversely, Sweden which has a higher GDP *per capita*, showed higher nominal prices, but PPP-adjusted prices appeared lower, suggesting relatively greater affordability. These findings are consistent with previous research (Wouters et al., 2025), which showed that results may differ depending on whether nominal or PPP-adjusted prices are used. That study reported that nominal drug prices tend to be higher in wealthier countries, whereas PPP-adjusted prices may be higher in poorer countries when affordability is considered.

The results of Vogler et al. (2015) show that Denmark has some of the highest nominal medicine prices, which aligns with our findings. However, after adjusting for purchasing power parity (PPP), Danish prices are not the highest among the analysed countries, suggesting that medicines are relatively more affordable for the population. In our study, both Denmark and Iceland had the highest nominal prices among European countries, but after PPP adjustment their prices were more moderate.

These findings highlight the importance of considering drug prices in the context of purchasing power parity. Nominal prices alone may provide misleading picture of affordability. By using PPP-adjusted prices, it becomes possible to better approximate the real economic burden associated with medicine costs and to identify countries where medicines may represent a relatively higher financial burden.

Our study also suggests that combining PPP-adjusted prices (Int$) with drug-specific indexes may provide a useful framework for cross-country comparison of pharmaceutical prices. While Int$ reflects the real cost of medicines after accounting for purchasing power, the drug-specific index offers an additional perspective by comparing the price-implied exchange rate with the actual PPP exchange rate. Applied to selected NOACs in our analysis, this approach illustrates how PPP-based metrics may help identify relative price positioning across countries.

Future research could extend this methodology to additional therapeutic areas and a broader set of countries to further explore international pharmaceutical price differences and their implications for medicine affordability. Such a combined PPP and drug-specific index framework could also help policymakers, researchers, and payers better understand cross-country price differences and identify where medicines may impose relatively higher economic burdens.

## Limitations

This study has several limitations. First, price data was collected during a specific timeframe for specific medications which may not fully reflect the price levels of other drugs across countries. Second, data for the United States were obtained, from community pharmacies, so they should be treated as illustrative of retail pharmacy price levels rather than as a fully representative national estimates. Importantly, analysis included prices based on 100% out-of-pocket payments, which means it does not consider reimbursement schemes or co-payments.

Despite these limitations, this analysis is a valuable source for gaining deeper insight into cross-country differences in drug prices. Further research incorporating net prices, reimbursement structures, and patient co-payments would enable a more comprehensive assessment of medication cost variation and market dynamics.

## Conclusion

The study found notable cross-country variations in the prices of the examined drugs. While some countries showed the lowest nominal prices, adjusting prices for PPP altered the relative price ranking, indicating that nominal prices alone may not accurately reflect real economic burden associated with drug purchasing (expressed in Int$). In contrast United States consistently represented the highest price market among analysed countries. The drug index analysis suggested that, relative to the benchmark, all examined currencies appeared undervalued.

Standardizing prices using purchasing power parity and expressing them in international dollars (Int$) allowed for more meaningful comparison across countries with different economic conditions. In addition, the drug-specific index provided a complementary perspective by assessing how national prices deviate from the level implied by PPP relative to a reference market.

Overall, the findings suggest the presence of substantial international disparities in PPP-adjusted selected NOAC prices. The use of international dollar standardization combined with drug-specific indexes provides a useful methodological approach for comparing drug prices across countries.

## Data Availability

The original contributions presented in the study are included in the article/Supplementary Material, further inquiries can be directed to the corresponding author.
