# Supplementary figures and images for: A new way to read the price: measuring global affordability of selected NOACs
Source: Front Pharmacol. 2026 Apr 29;17:1767958. doi: 10.3389/fphar.2026.1767958 (PMC13168899; doi:10.3389/fphar.2026.1767958)

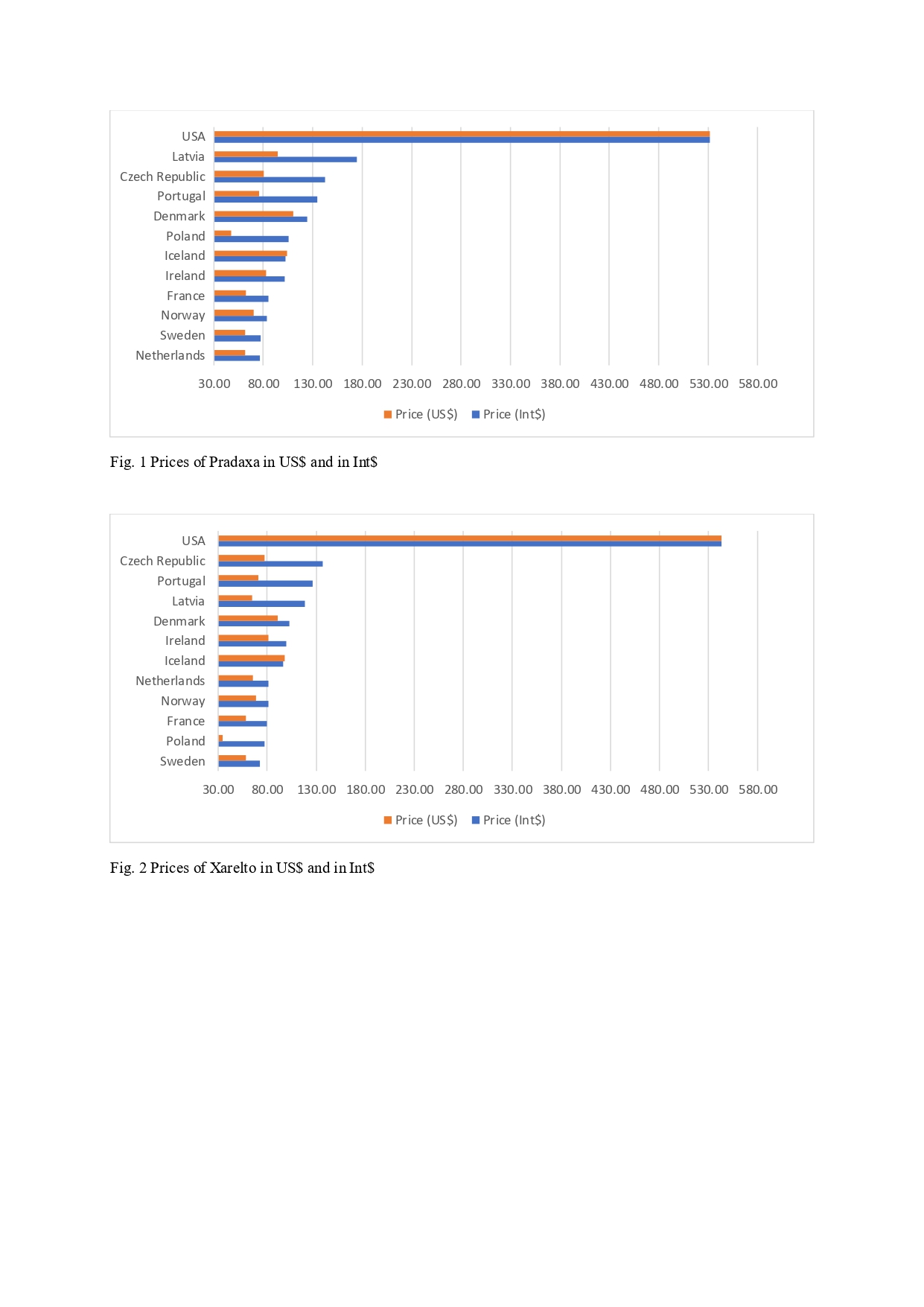

Supplement: Supplementary file 1 [file Image1.jpeg]

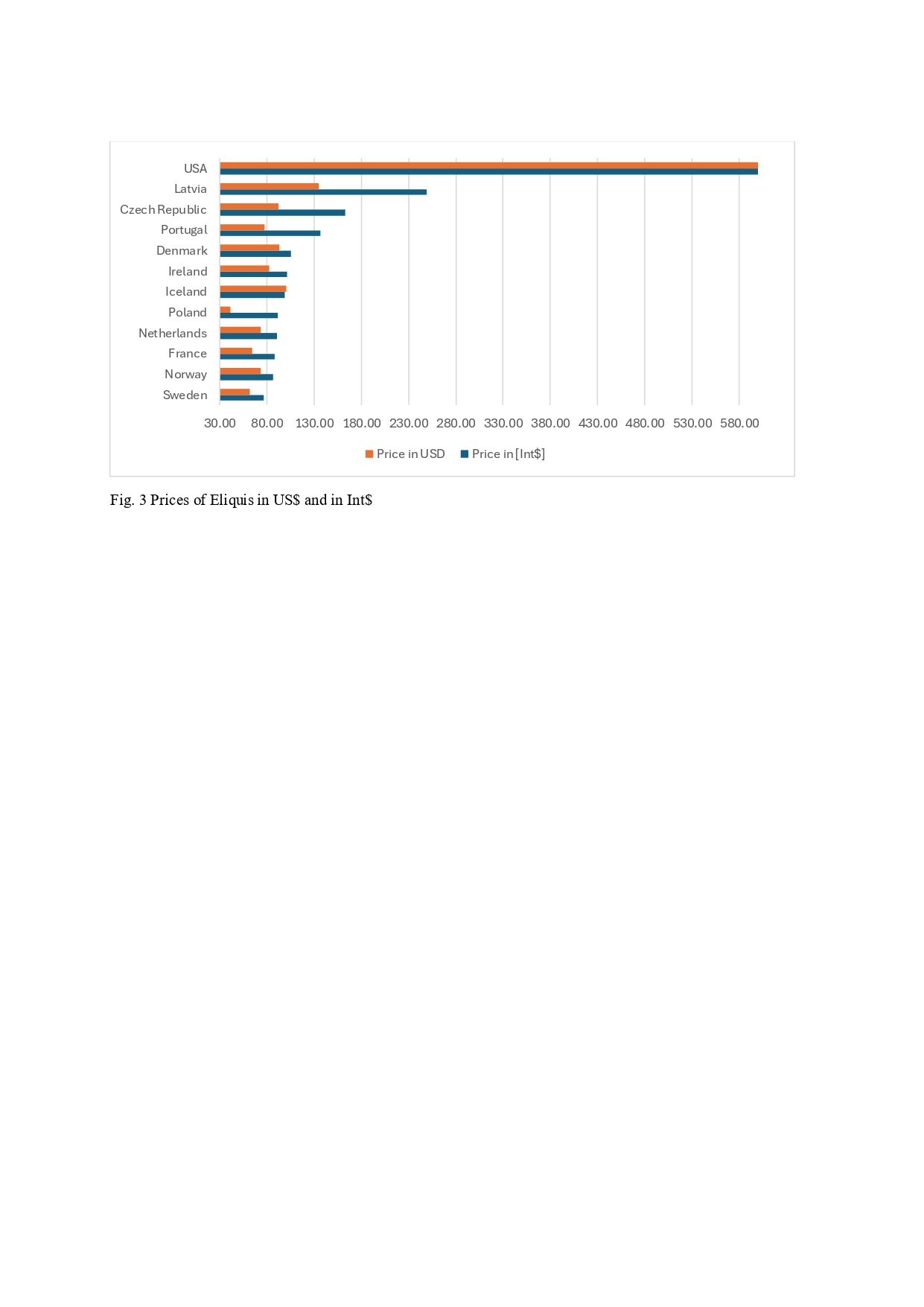

Supplement: Supplementary file 2 [file Image2.jpeg]
